# Detection of activated perforasome patterns and the correlation with acute internal load parameters during and following incremental cycling

**DOI:** 10.1007/s00421-025-05943-7

**Published:** 2025-09-11

**Authors:** Lukas Masur, Hanna F. Willenbockel, Thekla Cordes, Billy Sperlich, Peter Düking

**Affiliations:** 1 Department of Sports Science and Movement Pedagogy, Technische Universität Braunschweig, Pockelsstrasse 11, 38102 Brunswick, Germany; 2 Department of Bioinformatics and Biochemistry, Braunschweig Integrated Centre of Systems Biology (BRICS), Technische Universität Braunschweig, Brunswick, Germany; 3 Research Group Cellular Metabolism in Infection, Helmholtz Centre for Infection Research, Brunswick, Germany; 4 Integrative and Experimental Exercise Science & Training, Institute of Sport Science, University of Würzburg, Würzburg, Germany

**Keywords:** Individualization, Precision training, Muscle, Thermoregulation, Heat

## Abstract

**Purpose:**

Infrared thermography can be used to investigate the kinetics of thermoregulatory processes during exercise. The kinetics of the thermal contrast index (TCI), associated with activated perforasomes, and correlation with selected cardiovascular, metabolic, and subjective parameters have not been investigated during exercise.

**Methods:**

After a 5 min warm-up, *n* = 21 male cyclists performed an incremental test on a cycling ergometer until volitional exhaustion, followed by 15 min passive recovery. Lactate, heart rate, energy expenditure, oxygen consumption, and rating of perceived exertion were monitored continuously. Skin temperature (T_sk_) and TCI from anterior and posterior thigh were taken every 5 min following the TISEM checklist. TCI was calculated based on the temperature difference of the 10% hottest and 10% coldest pixels within the ROI.

**Results:**

TCI increased significantly in 27 of 36 total pairwise comparisons of consecutive exercise increments (*p* < 0.0001 to <0.05, ∆ 2.95 °C), while T_sk_ decreased in 20 increments (*p* < 0.0001 to <0.05, ∆ 1.85 °C) from rest to exhaustion. During the recovery period, TCI declined significantly at 5-min post-exercise (400 W: 4.33 °C vs. post 5: 2.93 °C, *p* < 0.0001), whereas T_sk_ increased significantly after 10 min (400 W: 30.10 °C vs. post 10: 31.33 °C, *p* < 0.01). TCI showed stronger positive correlations with the internal load parameter (*r* = 0.48 to 0.72, all *p* < 0.0001) compared to the inverse correlations of T_sk_ (*r* = −0.27 to −0.41, *p* < 0.0001).

**Conclusions:**

This study shows TCI reflects load changes more sensitively and correlates more strongly with physiological parameters than T_sk_, highlighting TCI’s potential as a real-time load surrogate.

## Introduction

Due to changes in climate, investigation of thermoregulatory aspects is of increasing relevance, e.g. since individuals with a low physiological capacity are at higher risk of heat-related hazards (Kenney et al. 2022). While thermoregulation is crucial for many populations to maintain optimal health (Byun et al. 2024), one population which is affected by changes in climate and where thermoregulation thereby becomes of increasing interest are physically active populations such as athletes, as the heat generated by active skeletal muscles imposes additional thermoregulatory challenges on the body. Particularly, prolonged or intense exercise causes a rise in body temperature, necessitating effective physiological responses to maintain homeostasis and prevent adverse health outcomes (Périard et al. 2021).

To investigate individual thermoregulatory processes and kinetics during exercise, infrared thermography (IRT) is increasingly used in physically active and athletic populations. IRT non-invasively quantifies skin temperature (T_sk_ [°C]) and visualizes surface thermal patterns (Arfaoui et al. 2014; Hillen et al. 2020). While IRT can be used to reveal different parameters, a lot of emphasis of previous research was placed on T_sk_, examining its changes in different regions of interest (ROI) of the upper and lower body (Korman et al. 2024; Merla et al. 2010; Duc 2015; Tanda 2016). During incremental exercise testing, T_sk_ decreases depending on the intensity due to vasoconstriction of the cutaneous arterioles and a redistribution of blood volume to fulfill oxygen requirements of the active muscles during exercise (Just et al. 2016). Beyond T_sk_, IRT also captures evolving thermal skin patterns, which can be representative of active perforasomes—vascular territories supplied by individual perforator vessels—which may facilitate heat dissipation by delivering blood from deep muscle layers to the skin surface in response to rising core temperature (Hillen et al. 2020; Merla et al. 2010). Underlying physiological considerations of T_sk_ and perforasomes activation variations mainly pursue two pathways pertaining on one hand to vasoconstriction and on the other hand to vasodilation of perforator vessels. Details of this mechanism are eloquently described in recent literature (Hillen et al. 2020).

To investigate perforasomes, different methodological approaches are currently discussed, including, e.g., entropy (Bogomilsky et al. 2022; Hu et al. 2025) and perforasome surface radiation pattern (PP_sr_) calculations (Hillen et al. 2020), the latter representing a thermal contrast index (TCI) within a ROI. TCI changes were previously associated with acute neural, cardiovascular, and thermoregulatory adaptations (Hillen et al. 2020), indicating its potential as a promising parameter for detecting physiological processes following intense exercise. Interestingly, the kinetics of TCI have not been quantifiably investigated. A case report in a single athlete noted a progressive increase in TCI during an incremental cycling test and proposed that this response may vary by individual fitness level, training status, or fatigue (Hillen et al. 2020).

The relationship between T_sk_ and physiological parameters has been assessed in previously published research. During exercise tests, T_sk_ was reported to correlate with lactate, heart rate, maximal oxygen uptake, and rating of perceived exertion (Chudecka and Lubkowska 2012; Korman et al. 2024; Hillen et al. 2023), indicating the simultaneous occurrence of thermoregulatory needs and individual internal load (Hillen et al. 2023). The correlation of TCI with cardiovascular, metabolic, and subjective parameters is currently not known. Physiological capacities are considered to impact thermoregulatory responses (Foster et al. 2020), highlighting the necessity to examine the relationship between internal load parameters and TCI. In addition, thermoregulatory load is considered to be one of the most limiting factors for performance outcomes during intense or prolonged exercise (Nybo et al. 2014). Elucidating the relationship between internal load parameters and thermoregulatory parameters could reveal information complementing established load monitoring approaches. An increased understanding of TCI kinetics and correlations with systemic physiological responses would enhance our insight into thermoregulatory responses to exercise and connections to other bodily systems. This knowledge could potentially allow to individualize training procedures, which becomes of special interest when thermoregulatory procedures are crucial, for example, in warm or hot environments.

However, to date, no study has systematically examined the acute kinetics of TCI or its associations with established cardiovascular, metabolic, and perceptual markers of internal load (Hillen et al. 2020; Korman et al. 2024).

Consequently, the aims of this study were to

(i) analyze TCI in conjunction with T_sk_ in response to increasing loads during and up to 15 min post an incremental exercise test
(ii) examine correlations of TCI and T_sk_ with selected cardiovascular, metabolic, and subjective parameters during and up to 15 min post an incremental exercise test

We hypothesize that there is a steady increase of TCI from resting conditions to volitional exhaustion, a subsequent decrease of TCI during the recovery period, and a negative correlation between TCI and T_sk_ as well as stronger correlations of TCI with the mentioned physiological parameters compared to T_sk_.

## Methods

### Participants

A total of 21 trained male athletes participated in the study. All participants were provided with detailed verbal and written information about the study procedures and gave written informed consent in accordance with the Declaration of Helsinki. The study was approved by the local ethics committee for research involving human subjects (approval number: D-2024-07).

In accordance with the Thermographic Imaging in Sports and Exercise Medicine (TISEM) checklist (Moreira et al. 2017) and recommendations of Fernandez Cuevas (Fernández-Cuevas et al. 2015), athletes were instructed to refrain from alcohol, smoking, caffeine, large meals 1 h prior to testing, ointments and cosmetics, showering 4 h prior to testing, physiotherapeutic applications or physical therapies, e.g., massages, heat and cooling therapies or electrostimulation, sunbathing, and strenuous physical exertion at least 24 h prior to testing. Participants were excluded if there were any signs of health disposition or injury.

### Procedures

#### Experimental design and exercise testing

**Fig. 1:**
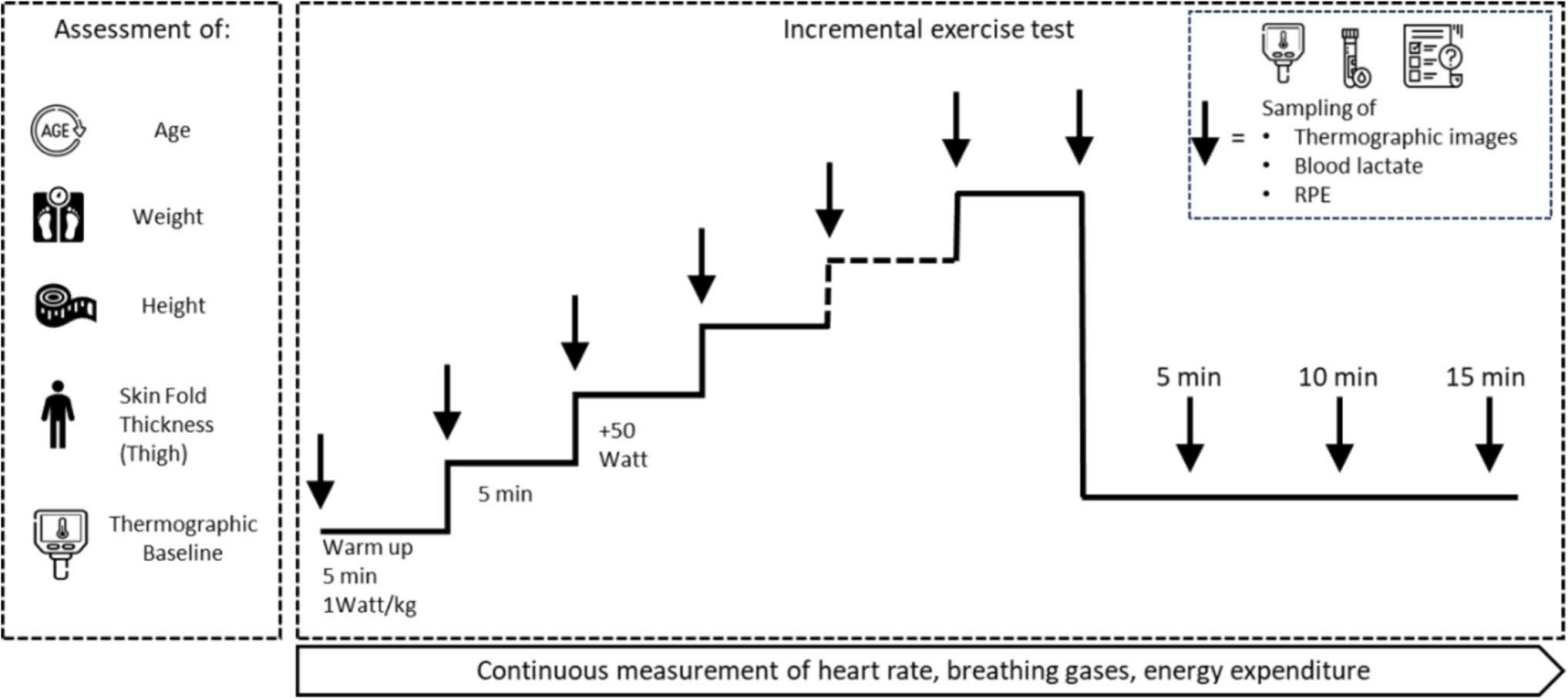
Schematic representation of experimental procedures. *RPE* Rating of perceived exertion

Experimental procedures are schematically illustrated in Fig. 1.

This study employed a cross-sectional design incorporating an incremental exercise test performed on a stationary cycle ergometer (Cyclus 2, RBM, Leipzig, Germany), equipped with an electromagnetic braking system and a power measurement accuracy of ±2%, as specified by the manufacturer.

#### Incremental exercise test protocol

The exercise protocol started with the warm-up period and an initial workload of 1 W per kg body mass for 5 min, proceeding with increments of 50 watts every 5 min until full volitional exhaustion. Participants were instructed to keep a pedal cadence of 80 revolutions per minute (rpm) throughout the testing and to remain seated during the test.

Maximal exertion and attainment of maximal oxygen uptake (V̇O_2max_) were confirmed when at least three of the following criteria were met: (i) a plateau in V̇O_2_ despite increased workload and ventilation; (ii) blood lactate concentration ≥9 mmol L⁻^1^; (iii) respiratory exchange ratio (RER) ≥ 1.10; and (iv) heart rate exceeding 95% of the age-predicted maximum.

#### Anthropometrics and physiological analyses

Prior to the incremental exercise test, anthropometric characteristics including body mass, body height, and body fat percentage were assessed. Body mass was measured using a bioelectrical impedance device (Tanita BC-601, Tokyo, Japan). Body height was determined using a standard measuring tape, and thigh skinfold thickness was estimated via the caliper method in accordance with established anthropometric protocols. Pulmonary gas exchange variables, including oxygen consumption (V̇O_2_) and carbon dioxide production (V̇CO_2_), were recorded using breath-by-breath analysis with a calibrated gas analyzer (Cortex Metamax 3B R2, Cortex Biophysik, Leipzig, Germany). Calibration was performed prior to each individual test using a certified calibration gas (15.8% O₂, 5% CO_2_ in N₂; Praxair, Düsseldorf, Germany) to match the expected physiological range of gas fractions. A precision 3-L calibration syringe was used to verify flow volume accuracy. According to the manufacturer, the oxygen sensor of the analyzer operates with a technical measurement error below 2% (Macfarlane and Wong 2012). Indirect calorimetry was conducted using the breath-by-breath gas analyzer (Cortex Metamax 3B R2, Cortex Biophysik, Leipzig, Germany), which calculates energy expenditure (EE) based on standard algorithms. For EE calculations, data points with percentage of V̇O_2max_ (%V̇O_2max_) values exceeding 85% were excluded, as estimates above this threshold are considered less valid. Heart rate was simultaneously monitored using the integrated telemetry system of the gas analyzer in conjunction with a validated chest strap monitor (Polar H10, Polar Electro Oy, Kempele, Finland). All calibration procedures were conducted following the manufacturer's specifications to ensure measurement accuracy and reliability. For the analysis of lactate with a handheld device (Lactate Pro 2, Arkray KDK, Kyoto, Japan) capillary blood was sampled from the right earlobe prior to the incremental testing, after each 5-min increment, immediately post-exercise, and at 5, 10, and 15 min during the recovery period. At the same timepoints, the Borg rating of perceived exertion (RPE; scale 6–20) (Scherr et al. 2013) was recorded. Athletes were instructed to self-assess their perceived exertion.

#### Thermographic measurement and analysis

The participants wore light sportswear not compressing adjacent tissue. For thermal image measurements and as used in other scientific articles (Rocha et al. 2022; Trovato et al. 2023, 2024; Krzysztofik et al. 2023), the uncooled FLIR E54 IR camera (Wilsonville, OR, USA) with noise-equivalent temperature difference <40 mK, 320 × 240 pixels of resolution and a temperature accuracy of ±2% was used.

Standardized thermal image recording was accomplished by the adherence of the TISEM checklist (Gomes Moreira et al. 2017) and recommendations of Fernández-Cuevas (Fernández-Cuevas et al. 2015). The camera was turned on at least 30 min prior to every measurement and placed perpendicular to the ROI using a tripod at a distance of 2.80 m from the participant. Average temperature and humidity were recorded on each testing day.

The participants acclimatized to room temperature and humidity for approximately 20 min prior to the test in a standing position. Thermal images of the anterior and posterior lower limb were taken pre, after each 5-min step, immediately after the test, and 5, 10, and 15 min into the recovery period.

**Fig. 2:**
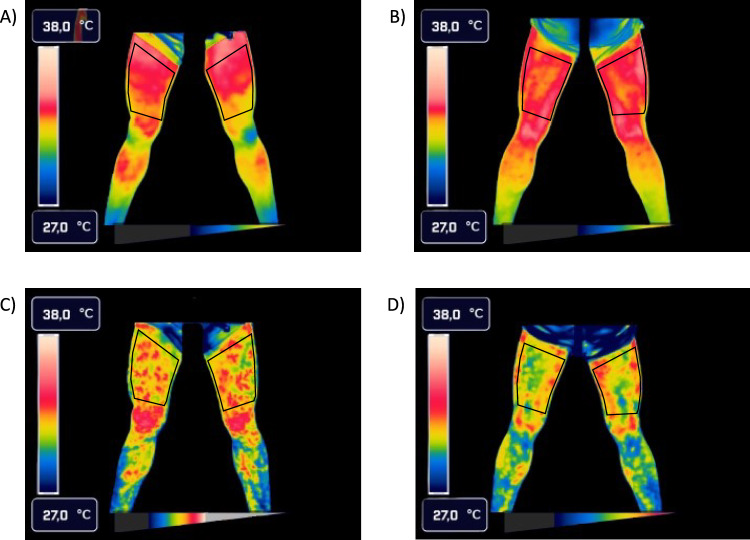
Thermographic images of the anterior (**A**, **C**) and posterior (**B**, **D**) view of lower extremities of an athlete prior to incremental testing (**A**, **B**) and immediately post full volitional exertion (**C**, **D**). Analyzed ROIs are delineated in every image

Thermographic analysis was performed with the approved software (Thermacam Researcher Pro 2.10 software, FLIR, Wilsonville, Oregon, USA), yielding the mean and standard deviation of T_sk_. The ROIs were defined based on anatomical criteria established in previous studies (da Silva et al. 2024, 2022), and are depicted in Fig. 2. The anterior and posterior thigh were delineated by drawing a straight line from the groin area toward the lateral side of the thigh, selecting the largest possible area bounded superiorly by the upper border of the patella (da Silva et al. 2022). After deriving temperature values from the pixels of the ROIs, TCI was calculated according to an equation previously applied in the literature (Hillen et al. 2020), originally termed PP_sr_. The calculation is based on the temperature difference of the 10% hottest and 10% coldest pixels within the ROI:

$${\mathrm{PP}}_{\mathrm{sr}}=10\% \text{darkest pixels}-10\% \text{lightest pixels within the region of interest}$$

Due to remaining validation studies, e.g., in conjunction with, e.g., Doppler method, we refer to this parameter as the TCI throughout this work.

#### Statistical analysis

A linear mixed-effects model examined the effect of increments on TCI, T_sk_, and internal load parameters (blood lactate, HR, V̇O_2_, EE, RPE), with increment as a fixed effect and random intercepts for each subject. The model was fitted using the lme4 package in RStudio (Bates et al. 2015). Pairwise comparisons between increments were performed using the emmeans package (Searle et al. 1980) based on estimated marginal means with Tukey’s adjustment for multiple comparisons. All pairwise comparisons were calculated using the estimated marginal means. The significance level for all statistical analyses was set at *p* < 0.05. To compare the sensitivity of TCI and T_sk_, the number of significant pairwise comparisons from pre to volitional exhaustion was summed for each parameter. Model assumptions (normality and homoscedasticity) were assessed using Q–Q plots and residual vs. fitted plots, respectively, following standard recommendations (Zuur et al. 2009; Fox and Weisberg 2019). No major violations were observed. Spearman’s rank correlation coefficients (r) were used to describe the relationship between internal load and thermographic parameters as well as between the thermographic parameters (T_sk_ and TCI). Correlation strength was defined as negligible (0.00–0.09), weak (0.10–0.39), moderate (0.40–0.69), or strong (0.70–0.89) (Schober et al. 2018). All statistical analyses were conducted using RStudio (version 2024.12.0, Posit Software, Boston, MA, USA).

## Results

A total of 21 athletes participated in the present study (all mean ± standard deviation; age: 28.1 ± 8.6 years, body mass: 76.5 ± 7.4 kg, body mass index: 23.0 ± 1.8 kg m^−2^, height: 182.6 ± 6.9 cm). The following physiological and anthropometrical parameters were measured before and during the test: V̇O_2_max: 58.9 ± 7.4 ml min^−1^ kg^−1^, maximal heart rate: 184.0 ± 10.9 bpm, and skinfold thickness thigh: 9.5 ± 3.5 mm. The mean ambient temperature and humidity were 23.8 ± 1.3 °C and 50.9 ± 0.1%, respectively.

Representative thermographic images from a single participant, captured during the pre-exercise condition and immediately following exercise cessation, are presented in Fig. 2. As no statistically significant differences were observed between the anterior and posterior ROIs for either T_sk_ or TCI during the incremental exercise test, all statistical analyses and results were calculated and reported for the overall thigh region.

### Kinetics of TCI and T_sk_ during the incremental exercise test and acute recovery

**Fig. 3:**
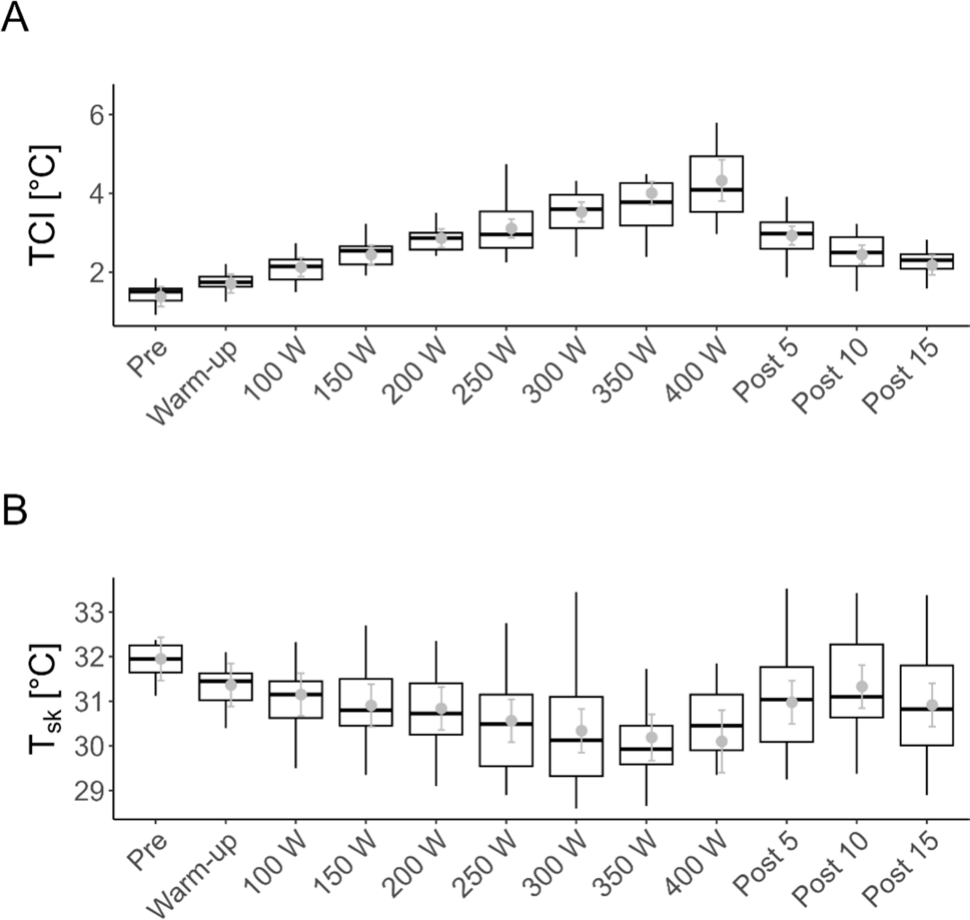
TCI (**A**) and T_sk_ (**B**) raw data (*black* boxplots showing individual observations, with the median, 25th, and 75th percentiles represented by the *box*; whiskers extend to the 1.5 IQR) and estimated marginal means (EMMs; *grey points*) for each incremental condition. EMMs were adjusted for the random effect of subject, with error bars representing the standard error. *TCI* thermal contrast index, *T*_*sk*_ skin temperature

Changes in TCI and T_sk_ throughout the incremental exercise test and subsequent recovery period are illustrated in Fig. 3. Raw data are depicted as black boxplots, while model-estimated marginal means derived from the linear model are illustrated in grey. There was a significant main effect of increments on T_sk_ values (F(11, 179.14) = 16.75, *p* < 0.001) and on TCI values (F(11, 184.59) = 52.89, *p* < 0.001).

All pairwise comparisons for the kinetics of TCI and T_sk_ throughout the incremental exercise test are presented in Table 1. The resting T_sk_ was 31.95 ± 0.24 °C, which progressively decreased during the exercise protocol. The first statistically significant decline in T_sk_ was observed from pre-exercise (31.95 ± 0.24 °C) to the warm-up phase (31.36 ± 0.23 °C) (*p* < 0.01). At exercise cessation, T_sk_ reached a minimum of 30.10 ± 0.35 °C. During recovery, T_sk_ increased to 31.00 ± 0.24 °C at 5 min post-exercise, with the highest post-exercise value occurring at 10 min (31.30 ± 0.24 °C).

**Table 1:** Estimated marginal means (EMMs) for TCI (°C) and T_sk_ (°C)

Step	*N*	T_sk_					TCI				
		EMM	SE	95% CI (lower)	95% CI (upper)	Significant difference to the closest previous step	EMM	SE	95% CI (lower)	95% CI (upper)	Significant difference to the closest previous step
Pre		31.95	0.24	31.46	32.44	n.a	1.38	0.13	1.13	1.63	n.a
Warm-up	21	31.36	0.23	30.88	31.84	Pre; *p* = 0.0098	1.71	0.12	1.47	1.95	n.a
100 W	21	31.15	0.23	30.67	31.63	Pre; *p* < 0.0001	2.13	0.12	1.89	2.37	Pre; *p* < 0.0001
150 W	21	30.90	0.23	30.43	31.38	Pre; *p* < 0.0001	2.44	0.12	2.21	2.68	Warm-up; *p* < 0.0001
200 W	21	30.84	0.23	30.36	31.32	Warm-up; *p* = 0.0213	2.86	0.12	2.62	3.10	100 W; *p* < 0.0001
250 W	21	30.56	0.24	30.08	31.04	100 W; *p* = 0.0062	3.11	0.12	2.87	3.35	150 W; *p* = 0.0001
300 W	18	30.34	0.24	29.85	30.83	150 W; *p* = 0.0185	3.53	0.13	3.28	3.78	200 W; *p* = 0.0002
350 W	12	30.19	0.26	29.67	30.71	200 W; *p* = 0.0198	4.01	0.15	3.72	4.30	250 W; *p* < 0.0001
400 W	4	30.10	0.35	29.40	30.80	100 W; *p* = 0.0316	4.33	0.27	3.80	4.85	250 W; *p* = 0.0008
Post 5	21	30.98	0.24	30.50	31.46	350 W; *p* = 0.0013	2.93	0.12	2.69	3.17	400 W; *p* < 0.0001
Post 10	21	31.33	0.24	30.85	31.81	400 W; *p* = 0.0045	2.45	0.12	2.20	2.69	Post 5; *p* = 0.0192
Post 15	21	30.92	0.24	30.43	31.40	350 W; *p* = 0.0054	2.18	0.12	1.93	2.42	Post 5; *p* < 0.0001

*CI* confident interval, *EMM* estimated marginal mean, *N* sample size per exercise stage, *n.a.* not available, *SE* standard error, *TCI* thermal contrast index, *T*_*sk*_ skin temperature

For TCI, the resting (pre-exercise) value was 1.38 ± 0.13 °C, increasing significantly to 4.33 ± 0.27 °C at volitional exhaustion (400 W). The first significant rise in TCI was observed at 100 W compared to pre-exercise (*p* < 0.0001). Following peak exercise, TCI declined significantly from 4.33 ± 0.27 °C at 400 W to 2.93 ± 0.12 °C at 5 min post-exercise (*p* < 0.0001). This downward trend continued throughout recovery, reaching 2.18 °C at 15 min post-exercise (post 5 vs. post 15: *p* < 0.0001).

The absence of significant differences between 400 W and the immediately preceding workload stages may be attributed to the reduced sample size at 400 W (*N* = 4), which likely limited statistical power and contributed to increased variability.

### Kinetics of selected cardiovascular, metabolic, and subjective parameters during the incremental exercise test and acute recovery

There were significant main effects of increments on lactate concentration (F[11, 193.4] = 34.51, *p* < 0.0001), HR (F[11, 193.2] = 224.31, *p* < 0.0001), VO_2_ (F[11, 195.6] = 362, *p* < 0.0001), EE (F[10, 687.4] = 2637.70, *p* < 0.0001) and RPE (F[11, 194.2] = 177.43, *p* < 0.0001).

**Fig. 4:**
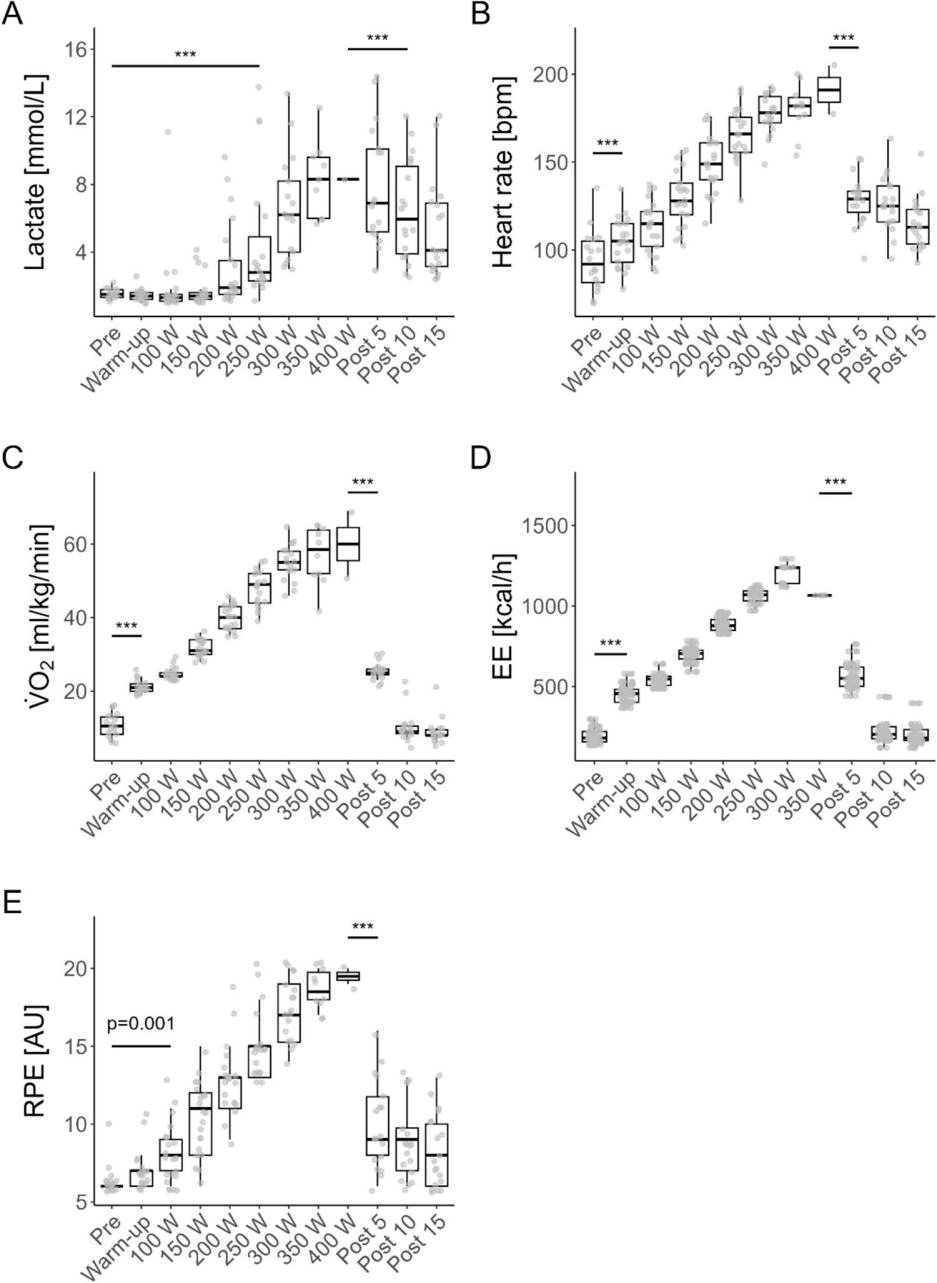
Kinetics of internal load parameters across exercise stages (**A** blood lactate, **B** heart rate, **C** oxygen consumption, **D** energy expenditure, and **E** RPE). *Grey dots* represent individual data points. *Black boxplots* show the median, interquartile range (25th, and 75th percentiles), and whiskers extending to the 1.5 interquartile range. *Horizontal lines* indicate the first significant difference from baseline or exhaustion, based on pairwise comparisons of estimated marginal means from a mixed linear model. ***p < 0.001. *EE* energy expenditure, *RPE* rating of perceived exertion, *V̇O*_2_ oxygen consumption, *W* Watt. For EE calculations, data points with percentage of V̇O_2_max (%V̇O_2_max) values exceeding 85% were excluded, as estimates above this threshold are considered less valid

The kinetics of every internal load parameter is shown in Table 2 and depicted in Fig. 4.

**Table 2:** Kinetics of internal load parameter across exercise stages

Step	*N*	Lactate (mmol/L)		Heart rate (bpm)		V̇O_2_ (ml/kg/min)		EE (kcal/h)^#^		RPE (AU)	
		EMM	SE	EMM	SE	EMM	SE	EMM	SE	EMM	SE
Pre	21	1.54	0.60	88.27	3.44	10.09	1.21	189.28	8.98	6.27	0.45
Warm-up	21	1.40	0.60	103.86	3.44	20.50	1.21	454.24	8.69	6.99	0.45
100 W	21	1.85	0.60	112.86	3.44	23.95	1.21	540.57	8.69	8.09	0.45
150 W	21	1.73	0.60	128.41	3.44	31.05	1.21	698.19	8.69	10.23	0.45
200 W	21	3.01	0.60	147.00	3.44	38.95	1.21	881.16	8.78	12.77	0.45
250 W	21	4.92	0.60	165.59	3.44	46.45	1.21	1060.13	9.37	15.18	0.45
300 W	18	7.29	0.63	177.87	3.52	53.35	1.26	1171.14	13.12	17.51	0.46
350 W	12	9.96	0.73	182.31	3.79	55.34	1.40	1067.10	26.09	19.38	0.51
400 W	4	12.21	1.21	185.18	5.32	59.44	2.16	n.a	n.a	20.70	0.79
Post 5	21	8.35	0.63	129.40	3.49	25.12	1.24	567.19	8.88	10.19	0.46
Post 10	21	6.43	0.63	128.45	3.49	10.32	1.24	225.14	8.88	9.09	0.46
Post 15	21	5.69	0.60	117.30	3.49	9.07	1.24	198.82	8.88	8.64	0.45

^#^Values > 85% of the %VO_2__max_ were excluded

*EE* energy expenditure, *EMM* estimated marginal mean, *N* sample size per exercise stage, *n.a.* not available, *RPE* rating of perceived exertion, *SE* standard error, *V̇O*_2_ oxygen consumption

### Relationship between TCI and T_sk_ and their associations with selected cardiovascular, metabolic, and subjective parameters

Statistical analysis showed a significant inverse correlation between T_sk_ and TCI over the incremental exercise test (*r* = −0.44, *p* < 0.0001). Correlation coefficients of the analysis between the investigated thermographic parameters and internal load parameters are summarized in Table 3. Internal load parameters and TCI were significantly positively correlated, with correlation coefficients ranging from *r* = 0.48 to 0.72 (*p* < 0.0001). T_sk_ showed significant negative correlations with internal load parameters (ranging from *r* = −0.27 to −0.41, *p* < 0.0001).

**Table 3:** Spearman’s correlation rho

Thermographic parameter	Blood lactate (mmol/L)	Heart rate (bpm)	V̇O_2_ (ml/kg/min)	EE (kcal/h)^#^	RPE (AU)
TCI	0.48***	0.72***	0.63***	0.58***	0.67***
*T*_sk_	−0.27***	−0.35***	−0.27***	−0.24***	−0.41***

***p < 0.0001

^#^Values > 85% of the %VO_2max_ were excluded

*EE* energy expenditure, *HR* heart rate, *RPE* rating of perceived exertion, *TCI* thermal contrast index, *T*_*sk*_ skin temperature, *V̇O*_2_ oxygen consumption

## Discussion

The aims of this study were to (i) analyze TCI in conjunction with T_sk_ in response to increasing loads during and up to 15 min post an incremental exercise test and (ii) examine correlations of TCI and T_sk_ with selected cardiovascular, metabolic, and subjective markers during and up to 15 min post an incremental exercise test. We hypothesized that there is a steady increase of TCI from resting conditions to volitional exhaustion, a subsequent decrease of TCI during the recovery period, and a negative correlation between TCI and T_sk_ as well as stronger correlations of TCI with the mentioned physiological parameters compared to T_sk_.

The main results of this study are:

1. From resting to volitional exhaustion, TCI increased significantly in 27 of 36 total pairwise comparisons of consecutive exercise increments (*p* < 0.0001 to <0.05, ∆ 2.95 °C), whereas T_sk_ values decreased significantly across 20 out of 36 comparisons (*p* < 0.0001 to <0.05, ∆ 1.85 °C), indicating that TCI may serve as a more sensitive parameter for detecting changes in responses to increasing external load during incremental exercise.
2. During acute recovery following volitional exertion, TCI values declined significantly after 5 min (400 W: 4.33 °C vs. post 5: 2.93 °C, *p* < 0.0001), and continued to decrease during the recovery period (Post 5: 2.93 °C vs. post 15: 2.18 °C, *p* < 0.0001), while T_sk_ significantly increased 10 min post-exercise (400 W: 30.10 °C vs. post 10: 31.33 °C, *p* < 0.01), indicating that TCI is more sensitive to cessation of exercise compared to T_sk_.
3. T_sk_ was negatively correlated (*r* = −0.27 to −0.41, *p* < 0.0001) with all physiological parameters assessed, whereas TCI indicated stronger positive correlations with all investigated parameters (*r* = 0.48 to 0.72, *p* < 0.0001), suggesting a higher sensitivity to respective cardiovascular, metabolic and subjective alterations.

### TCI changes with increasing load and during recovery

Our findings demonstrate significantly increasing TCI values from Pre (1.38 ± 0.13 °C) across exercise stages. TCI peaked at 400 W (4.33 ± 0.27 °C), with significant differences between most consecutive exercise increments (27 of 36 total pairwise comparisons of consecutive exercise increments; e.g., pre vs. 100 W and 200 W vs. 300 W, both *p* < 0.0001), indicating an intensity-related effect. Following peak load, TCI declined significantly post-exercise (400 W: 4.33 °C vs. post 5: 2.93 °C, *p* < 0.0001) and continued to decrease during recovery (post 5: 2.93 °C vs. post 15: 2.18 °C, *p* < 0.0001).

Thermographic research has referred to the appearance of hot spots, hot-spotted thermal patterns, or hyperthermal spots -sometimes described as tree-shaped pattern- without analyzing the underlying kinetics of perforasomes. The description of visible increasing perforasomes has been performed in different sports, such as judo, running exercise, or cycling tests (Brito et al. 2020; Ludwig et al. 2016; Merla et al. 2010). For example, 13 judokas developed hot spots over 70% of the entire body after a specific judo training (Brito et al. 2020). While judokas who developed hot spots showed lower body skin temperature compared to judokas without this thermal pattern (33.0 ± 0.4 vs. 33.6 ± 0.8 °C, *p* < 0.016), the study did not quantify the hot spots (Brito et al. 2020). Similarly, in *n* = 7 elite athletes, visual analysis showed a hot-spotted thermal pattern over the anterior thigh skin surface, and it was argued that this pattern strongly influenced skin temperature measurements due to the uneven thermal distribution in the selected ROI, but no quantification of the hot-spotted thermal pattern was performed (Ludwig et al. 2016). While consequently, the appearance of *hot-spotted thermal patterns* during different sports activities is not new, the present study is the first to quantify patterns. The findings of this study reveal a distinct increase in TCI during an incremental exercise test exhibiting important insights into the thermoregulatory processes of bodily systems besides the reduction of T_sk_.

Only one descriptive single-case study investigated TCI during the recovery period and reported a stable TCI of the thigh 5 min after an incremental cycling test (Hillen et al. 2020), contrasting our findings of a significant post-exercise decline after the same recovery period. Differences between this study and the existing single-case descriptive study remain elusive but could be due to inter-individual differences, sample size, or selected ROIs.

Underlying physiological mechanism resulting in TCI changes are not fully elucidated, but could be due to a rise in core body (or muscle) temperature (Merla et al. 2010; Ludwig et al. 2016). For example, it was argued that changes in TCI are a result of vasodilatation to reduce heat in muscles (Hillen et al. 2020). The TCI, based on PP_sr_ calculations (Hillen et al. 2020), is assumed to represent perforator vessels that extend perpendicular from musculature and fat tissue to the subdermal and dermal plexus (Saint-Cyr et al. 2009) which become visible in thermographic images as a tree-shaped pattern in response to increasing core temperatures (Arfaoui et al. 2014; Merla et al. 2010). Perforator vessels function to redirect blood leading to heat dissipation in the form of convection and vascular conduction to the skin surface (Hillen et al. 2020). In addition, cutaneous vasodilation—potentially related to TCI reflecting redistribution of blood through perforator vessel to the skin (Saint-Cyr et al. 2009; Kellogg et al. 1991aa) assessed via laser–Doppler flowmetry showed to be elevated in response to core body temperature increases of 0.2–0.3 °C (Kellogg et al. 1991b). While these results are an indication of the relationship between core body temperature and cutaneous vasodilation, future studies need to investigate the relationship between core body temperature and/or muscle temperature in greater detail. Potentially, TCI assessed via IRT could be used as a surrogate marker of core body or muscle temperature, easing their assessment due to the low cost and non-invasive nature of IRT.

### T_sk_ changes with increasing load and during acute recovery

Our findings revealed a decline in T_sk_ values across the incremental exercise protocol (pre: 31.95 °C to 400 W: 30.10 °C). Several of the reductions in T_sk_ were statistically significant (20 out of 36 total pairwise comparisons of consecutive exercise increments)—not only between resting and volitional exertion, but also between intermediate increments, such as 100 and 350 W (*p* < 0.0001), highlighting the effect of exercise intensity on T_sk_.

Our results align with previous research examining T_sk_ of the lower limbs during an incremental exercise test and in the acute recovery period. Research examined various sports and different performance levels, with all showing a decline in T_sk_ (Korman et al. 2024; Tanda 2016; Ludwig et al. 2016). Herein, T_sk_ results indicated a load-dependent vasomotor response, as higher load exhibited a pronounced decline of T_sk_ compared to lower load (Korman et al. 2024; Tanda 2016). Concerning the acute recovery phase, our results are in line with existing research, showing a significant increase in T_sk_ after 10 min (*p* = 0.0045) in the recovery period. Comparable studies reported slightly earlier increases, with elevations observed between 3 and 6 min (Tanda 2016; Ludwig et al. 2016). Apart from lacking statistical comparisons, these studies included a smaller sample size and used different ROI delineations. From a physiological perspective, the T_sk_ decline during incremental exercise was attributed to increasing intensity and corresponding sympathetic activation, which induces cutaneous vasoconstriction and redirects blood flow toward active musculature to meet elevated oxygen demands (Hillen et al. 2020). In summary, our results are consistent with prior studies, reinforcing the observation that exercise induces a progressive decline in T_sk_, reflective of sympathetic vasomotor regulation, with recovery trends returning T_sk_ toward baseline values post-exercise.

While T_sk_ shows significant increases from rest to exhaustion, aligning with previous research, this study provides additional insight by demonstrating that TCI is a more sensitive parameter for increasing loads, as more total consecutive exercise increments are significant.

### Correlation between TCI and T_sk_

There was a moderate inverse correlation (*r* = −0.44, *p* < 0.0001) between T_sk_ and TCI throughout the incremental exercise test and recovery. This finding supports existing observational research that sympathetic vasoconstriction and neurogenic vasodilation processes occur concomitantly during increasing external load (Hillen et al. 2020). Early studies suggest that cutaneous vasoconstriction and vasodilation compete due to thermoregulatory demands (Kellogg et al. 1991a). An additional notable finding is the temporal delayed onset of TCI compared to T_sk_. While T_sk_ showed a significant difference between Pre and warm-up (*p* = 0.0098), TCI demonstrated a significant difference only between Pre and 100 W (*p* < 0.0001). This is in line with previous observational research assuming a local delay of reflex neurogenic vasodilation over active muscles (Hillen et al. 2020).

In summary, our data suggest a shift in the primary physiological drivers throughout incremental exercise. Initially, T_sk_ reflects the body’s effort to redirect blood flow to meet oxygen and metabolic demands of active muscles. As exercise intensity increases, rising temperatures may become a more dominant factor, with TCI proving to be a more sensitive parameter for load increases - potentially reflecting the growing need for heat dissipation.

### Correlation of TCI and T_sk_ with cardiovascular, metabolic and subjective parameters

There were significant positive correlations between TCI and all assessed cardiovascular (HR, *r* = 0.72, *p* < 0.001), metabolic (V̇O_2_, *r* = 0.63, *p* < 0.001; blood lactate, *r* = 0.48, *p* < 0.001; EE, *r* = 0.58, *p* < 0.001) and subjective parameters (RPE, *r* = 0.67, *p* < 0.001).

Given its stronger correlation with cardiovascular parameter (e.g., heart rate) than with metabolic (e.g., blood lactate, V̇O₂, and energy expenditure) or subjective parameters (e.g., RPE), TCI appears to primarily reflect cardiovascular rather than metabolic or subjective load. Direct comparison with existing literature is not feasible, as this is the first study to quantitatively assess TCI in relation to cardiovascular, metabolic, and subjective parameters. Future research should further explore these associations across diverse populations and environmental conditions. Nonetheless, our findings highlight the potential of TCI as a non-invasive indicator of thermoregulatory responses and its link to cardiovascular and metabolic load during exercise.

Concerning T_sk_, our findings identified a significant negative correlation with cardiovascular (HR, *r* = −0.35, *p* < 0.001), metabolic (V̇O_2_, *r* = −0.27, *p* < 0.001; blood lactate, *r* = −0.27, *p* < 0.001; EE, *r* = −0.24, *p* < 0.001) and subjective parameters (RPE, *r* = −0.41, *p* < 0.001). These findings are consistent with previously published research. Studies analyzing cycling exercise tests reported slightly stronger significant negative correlations between T_sk_ of different ROIs(e.g., calf, *vastus lateralis* and *gastrocnemius medialis*) and internal load parameters such as HR, RPE and V̇O_2_ (Hillen et al. 2023; Duc 2015). These discrepancies may be attributed to the smaller sample size in the previous studies and differences in ROI selection, which have previously been shown to influence T_sk_ measurements (Hillen et al. 2020; Priego Quesada et al. 2016).

Collectively, the higher correlations of TCI with the selected cardiovascular, metabolic, and subjective parameters suggest that TCI may better reflect physiological load during and after incremental exercise compared to T_sk_.

## Strength, limitations and future research

This study is, to the best of our knowledge, the first to quantitatively investigate the kinetics of TCI during exercise and to correlate it with selected cardiovascular, metabolic, and subjective parameters. While it was the first study to investigate this parameter, and due to the specific protocols to measure TCI using IRT, we performed this study indoors under controlled conditions. Future studies should analyze the reliability of TCI in detecting activated perforasomes following acute exercise and develop a methodology to assess TCI outdoors under real-life conditions and during long-duration exercise during training and competition. While the incremental cycling test allowed us to analyze different intensity levels, outcomes of higher intensity increments may have been influenced by the test duration and not solely by the increased intensity. Therefore, results at higher intensities should be interpreted with caution. In addition, future studies could aim to evaluate the response of TCI with other parameters, such as core body temperature. Potentially, TCI could be used as a surrogate marker of core body temperature, which would ease its assessment due to the non-invasiveness of infrared thermography. The concomitant investigation of TCI and other parameters accessible by IRT, such as temperature entropy calculations in relation to physiological processes, should be considered in future research to establish a complementary approach for perforasome detection and to elucidate a more comprehensive understanding of relationships between existing perforasome patterns and physiological parameters.

## Conclusion

Investigations of thermoregulatory responses are getting increasingly relevant due to change in climate and in physically active populations, where heat generated by muscles imposes additional thermoregulatory challenges to maintain homeostasis. This study demonstrates that the TCI, measured via infrared thermography, is more sensitive to load changes during incremental cycling exercise and correlates stronger to selected cardiovascular, metabolic, and subjective parameters compared to T_sk_. While future research is necessary, this study indicates that heat dissipation reflected by TCI is more sensitive to increasing load compared to T_sk_, potentially reflecting the body’s response redistributing blood to active muscles to meet metabolic demands. These findings highlight TCI’s potential as a surrogate for cardiovascular and metabolic load. While more research is required, adoption of TCI assessment potentially could enhance precision monitoring of heat stress and inform individualized training and safety strategies.

## Data Availability

Data are available from the corresponding author upon reasonable request.

## Abbreviations

EE: Energy expenditure
EMM: Estimated marginal means
HR: Heart rate
IRT: Infrared thermography
ROI: Region of interest
RPE: Rating of perceived exertion
TCI: Thermal contrast index
TISEM: Thermographic Imaging in Sports and Exercise Medicine
T_sk_: Skin temperature
V̇O_2_: Oxygen consumption
V̇O_2max_: Maximal oxygen uptake
%V̇O_2max_: Percentage of V̇O₂_max_
V̇CO_2_: Carbon dioxide production
